# The Role of the Precuneus in Human Spatial Updating in a Real Environment Setting—A cTBS Study

**DOI:** 10.3390/life12081239

**Published:** 2022-08-15

**Authors:** Milos Dordevic, Sonja Hoelzer, Augusta Russo, José C. García Alanis, Notger G. Müller

**Affiliations:** 1 Department of Chronic and Degenerative Diseases, Faculty of Health Sciences (FGW), Potsdam University, 14476 Potsdam, Germany; 2 Department of Neuroprotection, German Center for Neurodegenerative Diseases (DZNE), 39120 Magdeburg, Germany; 3 Department of Neurology, Otto-von-Guericke University, 39120 Magdeburg, Germany; 4 Analysis and Modeling of Complex Data, Johannes Gutenberg University, 55122 Mainz, Germany; 5 Clinical Child and Adolescent Psychology, Philipps University, 35037 Marburg, Germany

**Keywords:** precuneus, spatial updating, TMS, cTBS

## Abstract

As we move through an environment, we update positions of our body relative to other objects, even when some objects temporarily or permanently leave our field of view—this ability is termed egocentric spatial updating and plays an important role in everyday life. Still, our knowledge about its representation in the brain is still scarce, with previous studies using virtual movements in virtual environments or patients with brain lesions suggesting that the precuneus might play an important role. However, whether this assumption is also true when healthy humans move in real environments where full body-based cues are available in addition to the visual cues typically used in many VR studies is unclear. Therefore, in this study we investigated the role of the precuneus in egocentric spatial updating in a real environment setting in 20 healthy young participants who underwent two conditions in a cross-over design: (a) stimulation, achieved through applying continuous theta-burst stimulation (cTBS) to inhibit the precuneus and (b) sham condition (activated coil turned upside down). In both conditions, participants had to walk back with blindfolded eyes to objects they had previously memorized while walking with open eyes. Simplified trials (without spatial updating) were used as control condition, to make sure the participants were not affected by factors such as walking blindfolded, vestibular or working memory deficits. A significant interaction was found, with participants performing better in the sham condition compared to real stimulation, showing smaller errors both in distance and angle. The results of our study reveal evidence of an important role of the precuneus in a real-environment egocentric spatial updating; studies on larger samples are necessary to confirm and further investigate this finding.

## 1. Introduction

Spatial updating plays an important role in everyday life. When we move through an environment this at first sight does not seem challenging—however, our brain must constantly and automatically update our egocentric mental representations of surroundings when visual, vestibular, kinesthetic, and/or proprioceptive signals indicate self-motion [1,2,3,4].

Spatial updating requires the perception of initial spatial positions of external objects and the construction of corresponding internal representations. Earlier studies show that such locational cues form the basis of an egocentric map of the environment that critically depends on the precuneus and connected inferior and superior parietal areas [5,6]. The precuneus is a highly interconnected associative cortical area in the posterior parietal lobe, and is involved in a wide spectrum of highly integrated tasks such as episodic memory retrieval, visuospatial imagery, and self-processing operation [6,7,8,9,10]. Moreover, the precuneus has dense connections to the premotor cortex [11] that can provide spatial information needed to organize actions such as walking, reaching and pointing. The precuneus and the posterior parietal cortex (PPC) are thought to be involved in visuospatial functioning [8,12,13,14]. Studies using patients with lesions to the PPC and precuneus showed a deceased spatial updating ability [15,16]. A more recent study by Weniger et al. [17] found that participants with parietal cortex lesions were strongly impaired in a virtual maze task (egocentric task), compared to control participant; in addition, volumes of the right-sided precuneus of lesioned subjects in this study were significantly related to performance on the virtual maze task. However, their performance on the virtual park (allocentric task) was entirely normal, indicating that the PPC is essential for updating in egocentric but not in allocentric space. Other studies found similar lateralization effect, with the right precuneus being associated with spatial processing [18]. Schott et al. [19] identified the precuneus as a key brain structure in the acquisition of detailed visuospatial information in a parieto-occipito-temporal network. In addition, medial parietal cortex is considered critical for self-motion perception [20]. According to the neural model of spatial memory by Byrne and colleagues [5] that is based on both human and animal findings, short-term egocentric representations reside in the precuneus. Moreover, they also hypothesized that updating occurs during self-motion. This was reinforced by Wolbers et al. [21] who used fMRI to show that only the precuneus remains significantly active during an egocentric updating task in a virtual environment that simulated forward self-motion, compared to a static condition. However, self-motion was simulated with an expanding optic flow field. The same is true for the study of Müller et al. (2018) [22] which used continuous theta-burst stimulation (cTBS) to show that the precuneus is crucial for spatial updating during visually simulated movement. TBS protocols have been widely used to induce plasticity, virtual lesions, and therapeutic effects in clinical trials due to their low stimulation intensity and short duration (cTBS < 1 min, compared to rTMS 20–30min), which make them easy to apply and more comfortable for subjects [23,24]. In addition, previous studies have shown that inhibitory cTBS lasts for approximately 30 min after stimulation [25,26], allowing for offline testing conditions. However, spatial updating in the real world involves not only visual but nearly all sensory systems including vestibular and proprioceptive ones [27]. Hence, with the recent VR-experiments which only applied optic flow to simulate self-motion, little is known about egocentric spatial updating in real-life settings—i.e., conditions whereby participants are actually moving through an environment instead of observing optic flow while being static. In addition, there is currently no reliable real-environment paradigm with high sensitivity and specificity values—determined using the region of interest (ROI) analysis [28]—which can be useful for assessment of the spatial updating ability and, correspondingly, precuneus integrity.

Thus, in this study we investigated the impact of inhibiting the right precuneus in a real environmental egocentric spatial updating paradigm. A real environmental setting was chosen to provide multisensory self-motion cues. The inhibition on the precuneus was induced by continuous theta-burst stimulation, which was successfully applied by previous studies [22,25,26,29]. We hypothesized that inhibition of the precuneus through cTBS will lead to worse performance on egocentric spatial updating paradigm in a real environment compared to sham condition. Thus, the aim of this study was to test if egocentric spatial updating in real environment is mediated by the right precuneus.

## 2. Materials and Methods

### 2.1. Ethical Approval

This study was approved by the Ethics Committee of the Medical Faculty at the Otto von Guericke University (approval number: 49/14). All participants gave written informed consent in accordance with the Declaration of Helsinki.

### 2.2. Participants

Twenty healthy young participants (9 females, mean age 23.00 ± 2734 years, ranging from 18 to 29) were recruited among students at the University in Magdeburg by means of public advertisement. All participants reported normal or corrected-to-normal vision, and all were right-handed. According to a self-report, they were without any known contraindications to cTBS. Participants received monetary compensation and gave written informed consent to participate in the experiment. The experiment took place in the DZNE Magdeburg in the period from January 2019 to December 2019.

### 2.3. Study Design

This study was organized as a within subject cross-over design. Each participant underwent two sessions on two nonconsecutive days (separated by at least 3 days), with real cTBS-stimulation (stimulation condition) and with sham cTBS-stimulation (sham condition, coil turned upside down). Stimulation and sham conditions were randomized using randomization software (https://www.randomizer.org accessed on 15 January 2019).

On both occasions participants received a short training trial (not taken into account for the analysis) followed by either sham or real stimulation and by the spatial updating task lasting about 20 min. Before stimulation (or sham) all participants underwent a static (control) simplified session of trials, whereby there were asked to memorize and walk to a specific target location—this control condition, which did not involve spatial updating, was used in the analysis and the purpose was to exclude factors such as difficulties while walking blindfolded, vestibular or working memory deficits.

### 2.4. Spatial Updating Paradigm

**Figure 1:**
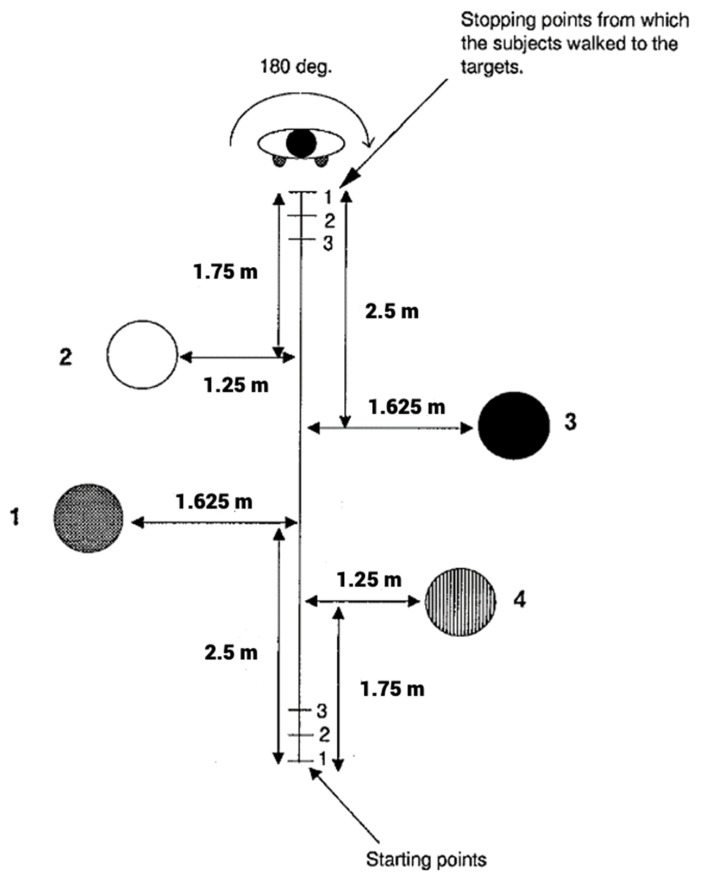
Experimental setting showing the path (start to stop) participants were walking with open eyes as well as the targets (circles numbered 1–4) they were supposed to memorize and subsequently reach by walking to them blindfolded.

We used a modified version of the experimental setting published by Farrell and Thompson (1998) [2] (Figure 1). The room size used for the experiment was 12 m long and 8 m wide. Three starting points (0m, 0.5m and 1m from the first starting point) and three stopping points (5 m, 5.5 m and 6 m from the first starting point) were marked on the floor for the experimenter, but were not clearly visible for the tested person, since the markers were placed under the walking line and were thus difficult to recognize—we verified that participants did not notice these markers. Differently colored flat circular targets (diameter 15 cm) were placed on predetermined locations (see Figure 1), and provided no tactile cues to participants (setting 1: dark blue, yellow, orange, green; setting 2: sky-blue, red, white, violet). The targets were placed so that target objects’ positions were symmetric between starting and stopping points. The experimental setting remained the same for both visits to the testing center (once for sham and once for stimulation), with the exception that the circular target objects were placed on mirrored locations and their colors were varied to prevent a learning bias. The order of the two conditions was also counterbalanced.

The experiment included an updating and a control condition as follows:

(a) Static (control) condition: The participant is first allowed to memorize locations of the four targets from a starting point. He/she is then blindfolded by the examiner and asked (by naming its color) to walk directly to one of the targets, where his/her ending position is marked on the floor. The time participants were allowed to memorize the targets is approximately identical as in the updating condition (5 s). That is, approximately the same time needed to reach the stopping points in the dynamic conditions was introduced before each target in the static condition was named (for the participant to begin walking to it). Thus, participants in this condition did not benefit from a shorter delay between encoding and retrieving (departure towards the target).
(b) Dynamic (updating) condition: The participant is walked to a starting point and is first allowed to memorize locations of all four targets from that starting point. He/she is then guided (with open eyes) by the experimenter to a respective stopping point in a straight line, using tactile (examiners held participants’ shoulders) and verbal instructions (“start/stop” walking). The movement velocity is kept as constant as possible. After reaching the stopping point the participant is blindfolded and is then turned by 180 degrees to face the starting point. Next the experimenter instructs the participant to which target he/she should walk by naming its color. After the participant has walked to the target, his/her position is marked on the floor for assessment of errors in distance (centimeters) and angle (degrees). Still blindfolded, the participant is then guided back to the next starting point for the succeeding trial. The participants were instructed to memorize the locations of circular objects in relation to their own body (egocentric).

In total, during each of their two visits to the testing center (once for sham and once for stimulation) every participant performed 24 trials—namely, 12 static and 12 dynamic trials (3 trials with different starting/stopping points times 4 targets each). For each trial, the order of presentation of target objects as well as starting and stopping points was randomized; likewise, as mentioned above, for each visit the colored targets were placed on mirrored locations with respect to the walking line, compared to their previous visit. In both conditions, the participants were asked to walk to target objects immediately after being instructed, without allowing them to think about where they should go, to make sure they rely on automatic updating.

Before each condition, the participants were given a short training in blind walking. After walking in a straight line, they were instructed to turn 180 degrees and walk to a specific target, in an order randomly chosen by the experimenter. Here, the participants were allowed to see where they had walked, and trials were continued until the participant was able to stop within one pace (approximately 60–70 cm) of the target. All participants achieved this within 5–20 trials.

### 2.5. cTBS

The spatial updating task was performed in two experimental sessions (sham and cTBS stimulation) in a counterbalanced order. Previous studies showed that the inhibitory cTBS lasts for about 30 min after the stimulation [25,26].

We applied continuous theta-burst stimulation (cTBS) according to the protocol previously used by [22,25]. cTBS was controlled by a MagPro stimulator (X100 + MagOption, MagVenture, Farum, Denmark), and pulses were delivered by a water-cooled figure-of-eight coil with an outer diameter of 75 mm (Cool B-65, MagVenture, Farum, Denmark). A cTBS train consisting of 267 bursts (801 single pulses) was applied over right PPC for 44s at 6 Hz, with each burst containing three biphasic pulses (repeated at 30 Hz). Pulse intensity was set to 100% of the individual resting motor threshold (MT), which was defined as the lowest intensity able to induce a motor evoked potential of 100 μV (recorded from the right abductor pollicis brevis) in at least 50% of a series of ten single pulses applied to the left motor cortex. Mean pulse intensity was 45.9% (ranging from 32% to 55%) of the maximal stimulator intensity for all participants. During stimulation, the coil was held with the position of maximal magnetic field tangentially on the participant’s skull by the examiner (controlled through Localite).

On the basis of spatial coordinates provided by Wolbers et al. (2008), the stimulation site was determined. Slightly different Talairach coordinates for the right precuneus were reported for the different experiments of that study, therefore, we averaged the values across experiments, resulting in the following coordinates: x = 5.33, y = −54.33, z = 47.33. The used navigator system (Localite TMS Navigator, version 2.1.18) enables co-registration of the individual’s scalp surface via optical tracking supported by infrared marks (Polaris, NDI medical, Waterloo, ON, Canada) and warps a MNI template brain to match the individual’s head. With that information the coil position and orientation on the scalp surface relative to the entered stimulation site coordinates were calculated by the system and monitored during stimulation. At control condition, we applied sham stimulation by turning the coil upside down so that the cTBS impulses did not reach the brain. Therefore, we were able to perform an offline cTBS application, and the experimental task was performed in the complete absence of any stimulation, which reduces the potential influence of unspecific effects [25,29]. Moreover, a sham noise generator (MagVenture, Farum, Denmark) was used in both conditions. Due to this, acoustic disturbance and vibrations of the coil were identical across both sessions.

### 2.6. Outcome Variables and Data Analysis

Pre-specified primary outcomes were distance to the target object (i.e., distance error in centimeters) and absolute angular deviation from target (i.e., angular error in degrees).

Data were analyzed in the R programming environment [30]. Data were analyzed using a linear mixed effects regression approach. These analyses were carried out using the lmer() function from the R-package *lme4* [31]. Like ordinary least squares (OLS) models, mixed effects regression examines the relationship between a set of predictors (e.g., stimulation condition) and a response variable (e.g., distance errors in cm). However, the repeated measures design (multiple measurements extracted from one subject) of our study might lead to strong interdependencies in the data, thus violating one of the key assumptions (the conditional mean should be zero) of OLS models [32]. A mixed-effects regression approach allowed us to account for individual variation of the response variable’s variance (e.g., more similar errors within subjects than between subjects), which, if led unaddressed, can lead to increased error variance in the OLS models, diminishing their validity and statistical power. All models were fitted by restricted maximum likelihood and estimated a random intercept for each subject (i.e., nesting observations within participants). Main effects and interactions were assessed via Type III Wald F-tests as implemented in R-package *car* [33]. Stimulation (sham vs. cTBS stimulation) and Movement (static vs. dynamic) were effect (i.e., deviation) coded prior to analyses and included in the model as fixed within-subjects categorical predictors). To test interaction effects, pairwise contrasts were computed based on the estimated marginal means using the R-package emmeans [34]. All p-values for the pairwise comparisons were adjusted according to the Bonferroni method. The significance level was set to α = 0.05 Table 1 shows the means ± standard deviations; in addition, effect sizes (Cohen’s *d*, calculated as: *d* = 2 × *t*/$\sqrt{N}$) and 95% confidence intervals of the estimated difference are reported. Effect size magnitude was assessed as follows: ≥0.2 indicated small, ≥0.5 medium and ≥0.8 large effects [35]. All datasets were checked for and met the assumptions of normal distribution and homogeneity of variance. Finally, Receiver Operating Characteristics (ROC) analysis was applied for determination of sensitivity and specificity values obtained from this paradigm, based on distance error values.

## 3. Results

Analyses revealed a significant main effect of stimulation condition on distance error (F(1, 57) = 6.705, *p* = 0.0122) but no significant main effect of stimulation condition on angular error (F(1, 57) = 1.005, *p* = 0.3205). As summarized in Table 1, participants performed significantly worse after cTBS stimulation than after sham condition (t(57) = −4.74, SE = 4.74), with larger distance errors following cTBS (M = 56.45, SD = 17.85) compared to sham (M = 8.44, SD = 4.35), but showed comparable performance in terms of angular error (t(57) = −1.00, SE = 0.64). Further, we found that distance errors were significantly greater during dynamic movement (M = 75.30, SD = 21.54) compared to the static protocol (M = 49.26, SD = 14.99; t(75) = −8.67, SE = 3.35). Similarly, angular errors were significantly greater during dynamic movement (M = 10.18, SD = 3.89) compared to the static protocol (M = 6.06, SD = 2.54; t(57) = −6.34, SE = 0.65).

**Figure 2:**
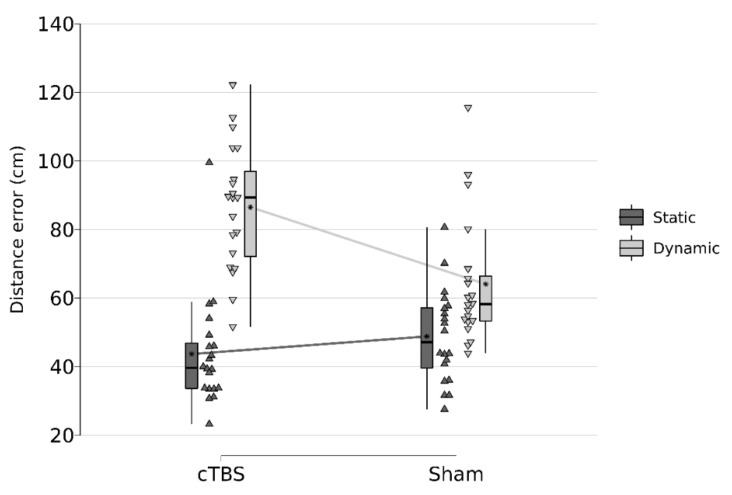
Interaction effect Stimulation * Movement on distance errors in centimeters.

**Figure 3:**
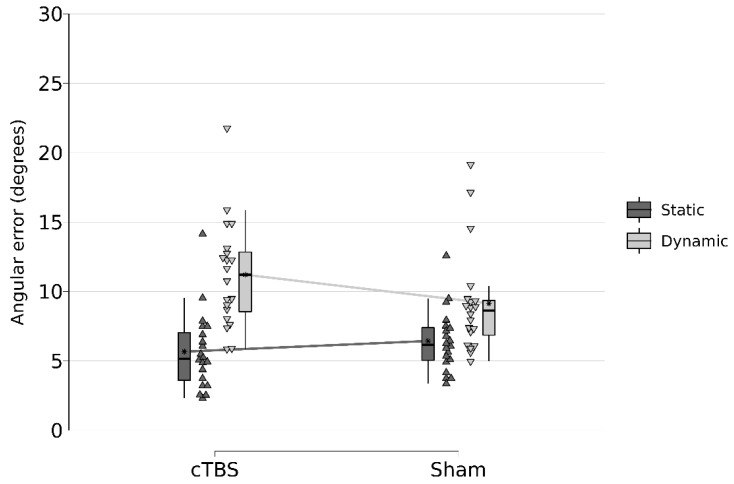
Interaction effect Stimulation * Movement on angular errors in degrees.

Importantly, these effects were further explained by a significant two-way interaction between stimulation and movement for distance errors (F(1, 57) = 16.963, *p* = 0.0001) (Table 2, Figure 2 and Figure 3). Pairwise comparisons revealed that, while there were significant differences between the static (M = 48.83, SD = 13.68) and dynamic (M = 64.07, SD = 18.57) movement protocols (t(57) = −3.12, SE = 4.74) after sham, this effect was more pronounced following cTBS stimulation (t(57) = −9.04, SE = 4.74). For angular errors, analyses revealed a similar two-way interaction between stimulation and movement for distance errors (F(1, 57) = 4.809, *p* = 0.0324). Pairwise comparison indicated that there were significant differences between the static (M = 6.44, SD = 2.24) and dynamic (M = 9.15, SD = 3.74) movement protocols (t(57) = −2.97, SE = 0.91) after sham, but similarly, this effect was more pronounced after cTBS stimulation (t(57) = −6.08, SE = 0.91).

Thus, cTBS stimulation impaired to subjects’ ability to walk exactly to marked and previously memorized points, represented by a higher angular deviation from the correct path towards marked points as well as by a larger distance from the point where they ended up to marked points. The effect sizes are provided in Table 2.

**Figure 4:**
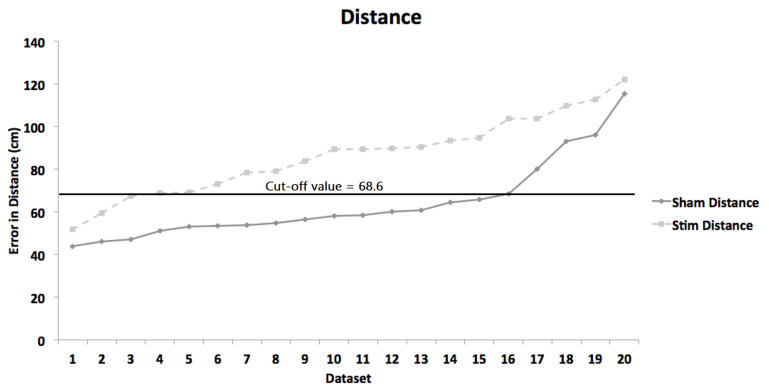
Error in distance for each participant at both levels of the stimulation condition (Sham and Stimulation) on dynamic trials.

**Figure 5:**
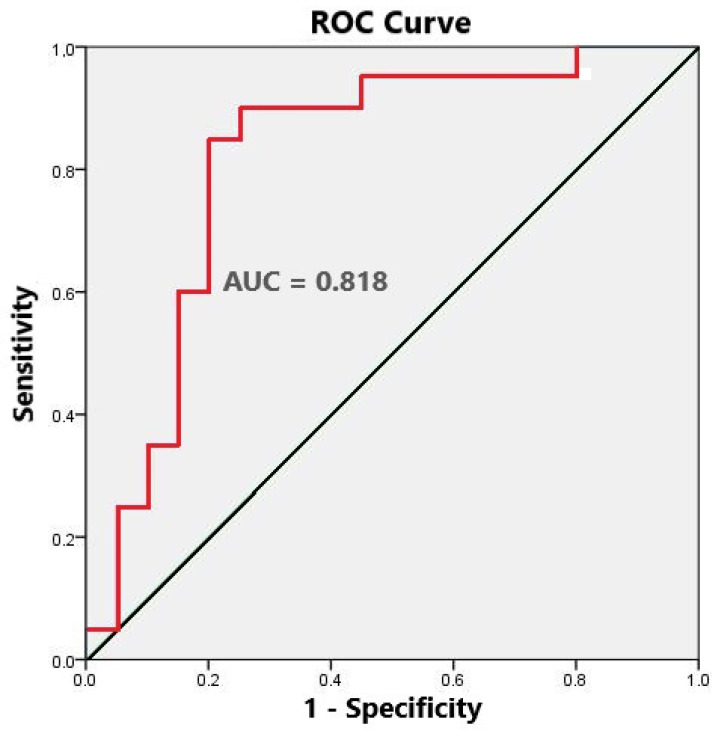
Receiver operator characteristic (ROC) curve (in red) for Sham vs. Stimulation condition (for distance parameter).

ROC curve is very informative method of establishing the sensitivity and specificity values of any test—to establish a cut-off value, or the value below which all of the stimulated participants’ values will be located, we plotted values for each participant in our study (sorted from lowest to highest error on sham and stim conditions), for both conditions (Figure 4). The cut-off value was then established by selecting the value at which the classification of participants in the two conditions was optimal (as calculated by ROC-curve in Figure 5). As displayed, the optimal established value was 68.6 cm. At this cut-off, 17 of 20 (85%) performances of stimulated participants were above the respective threshold, whereas only 4 of 20 (80%) performances of sham participants remained above this threshold. From these data, it appears that the error in distance has a good potential in differentiating between stimulated and sham conditions.

Figure 5 depicts the area under the curve (AUC) values from the receiver operator characteristic (ROC) analysis, as a comparison between the two conditions (Sham vs. Stim). For the cut-off threshold of 68.6 cm, the highest AUC-value was 0.818 (95% confidence interval 0.676–0.959). The corresponding sensitivity and specificity values were 85% and 80%, respectively.

## 4. Discussion

In this study our main hypothesis was confirmed—namely, inhibiting the precuneus through cTBS led to worse performance in an egocentric spatial updating paradigm in a real environment, when compared to a sham stimulation. Therefore, the obtained outcome allows us to speculate that the egocentric spatial updating ability is mediated by the right precuneus, at least in relation to the applied real-environment spatial updating paradigm. These effects pertain to participants’ reduced ability to recall both translational (distance) and rotational (angular) estimation of targets’ locations in response to cTBS stimulation of the precuneus. Moreover, since no significant difference could be found on static trials, the obtained results cannot be attributed to general deficits in navigational abilities or other potential confounds such as working memory. Finally, for the first time we could show that participants could be categorized into a stimulated and a sham condition, with relatively high sensitivity and specificity (85% and 80%, respectively) values, based on their distance errors only—this might provide a sound base for future diagnostic procedures and assessments of deficits in a real-environment egocentric spatial updating task.

The findings of our study are consistent with previous studies on participants with brain lesions, which also reported impaired egocentric spatial updating, resulting mainly from lesions in the posterior parietal cortex (PPC), but especially in the precuneus [15,17]. In the study by Aguirre and D’Esposito [16], the authors described patients with lesions to the superior parietal lobule who suffered from egocentric navigation deficits; namely, they could not define positions of objects and landmarks relative to themselves. Likewise, the study by Farrel and Robertson [15] on patients with lesions in these areas reported impaired non-visual updating of body-centered spatial relationships. A more recent study by Weniger and colleagues [17] assessed spatial memory in a large-scale virtual-environment in patients with unilateral parietal cortex lesions (due to infarction or intracerebral hemorrhage) and found significantly worse performance on the egocentric navigation task, but not the allocentric task, compared to healthy controls. Moreover, volumes of the right precuneus of the patients were significantly related to performance on the virtual maze, indicating better performance of patients with larger volumes. However, compared to studies on patients with lesions in these brain areas, by using cTBS we were able to create a more homogenous effect—namely, well-known effects of general cognitive and mnemonic disturbance as well as residual neurological symptoms, resulting from unspecific neural lesions, have been avoided in our study.

Given that spatial updating may not only rely on internal (e.g., vestibular and proprioceptive information) but also on external perceptual cues (e.g., optical flow), many studies investigated the role of PPC and precuneus in spatial updating using a virtual reality approach, with optic flow being the only sensory cue [17,21,22]. However, vestibular, proprioceptive and tactile cues generated by physical motions are generally considered to be essential and sufficient for automatic spatial updating [1,4,15,36,37]. Indeed, it has already been shown that the presence of a real environment corresponds with the best performance [38]; on the other hand, some studies found that optic flow alone appears to be insufficient for efficient spatial updating [3,39]. Moreover, Schöberl and colleagues [40] found a reduced activation of the prefrontal cortex (PFC) while participants were performing the VR-based tasks, compared to the real environment task—since the PFC is known to be crucial for planning and decision making (such as with path planning [41]) and considering that real space navigation in novel environments always requires decision making and planning processes directly linked to motion along novel routes, there could be a weakness of several VR settings, especially those where participants do not actually move (e.g., walk) and movement is only visually simulated (e.g., by optic flow), whereby frontal lobe activations are underrepresented. Thus, in our current study, by choosing a real-environment scenario, we were able to assess the effect on the spatial updating ability while relying on the most relevant cues during self-motion, including tactile, vestibular and proprioceptive

Although when navigating in familiar environments or over longer durations humans predominantly use an allocentric reference frame [42], we have several reasons to argue that our task was well-designed to assess egocentric and not allocentric spatial updating. Firstly, movement in unfamiliar environments, especially in smaller areas, requires a constant updating of relationships between the observer and each object in the visual field [42,43]. Given that the characteristics of our task (body motion in a real environment) correspond well with those conditions associated with egocentric spatial updating, it is reasonable to assume that self-motion cues were used to update the stored egocentric object representations. Secondly, participants were intentionally constrained during the task, since the experiment did not contain any landmarks and we chose an unfamiliar environment with small movements, so they had to complete it by relying on egocentric cues. Finally, participants themselves reported to have memorized egocentric cues during the experiment. Therefore, it is justified to assume that the impairments in the dynamic updating task in response to the inhibitory cTBS were predominantly caused by egocentric memory deficits. Nevertheless, it must be considered that a spatial navigation task cannot be pure egocentric or allocentric, rather a combination of both types of information in spatial navigation and learning is likely [44]. Additionally, in egocentric spatial updating, the updating efficiency is highly dependent on the number of objects one has to update—this is because of the fact that, as the observer moves, each of the given objects must be updated [43]. It was previously shown by Wolbers and colleagues [21] that humans can successfully update up to four spatial positions during self-motion, which was also the rationale behind choosing this number of objects in our study.

Relatively high sensitivity and specificity values obtained from this paradigm speak for its application in future studies, but also for investigating its potential for further development. The aim could be to develop a highly-predictive tool for the assessment of the spatial updating ability and, correspondingly, precuneus integrity—predominantly in relevant groups of patients, such as those suffering from stroke or dementia. For instance, the paradigm from this study could be implemented in testing patients suffering from mild cognitive impairment (MCI), often an early stage of dementia, since a link between precuneus deactivation and MCI has already been reported [45], even while atrophy is still not present in this brain region [46]. Moreover, other authors [47] argue that hypoperfusion starts from the precuneus, well before the onset of dementia, and spreads to the rest of the parietal cortex and the cingulate gyrus along with progression of AD. Furthermore, the precuneus dependent egocentric updating in MCI seems to be impaired in landmark-free virtual-reality maze, requiring the participants to use only the sequence of egocentric turns [48]. Additionally, other studies also indicated that navigational impairments occur before patients fulfill the diagnostic criteria for AD [49,50]. Therefore, in addition to assessment, our spatial task may be useful to differentiate between subjective cognitive impairment (SCI) and MCI and, moreover, detect early MCI and precuneus involvement. This paradigm can be also considered for various other clinical conditions, including those involving disorders of consciousness [51].

Our study also has several limitations that need to be mentioned. First, the inhibitory effects of cTBS were presumably not limited to the precuneus but may have extended to neighboring regions of the medial parietal cortex and beyond. However, this might not be as dramatic, considering the findings of earlier fMRI studies showing that the precuneus appears to be the brain region essential for spatial updating [21]. Second, even though we chose a real-environment setting, the design of the environment was very simplistic and artificial, with the target objects being only flat circles on the ground. However, although an even more realistic environmental stimuli would perhaps affect our results to some extent, we are convinced that including the actual multisensory self-motion remains the most relevant and influential component. Moreover, the simplification of environment was necessary to ensure participants would rely on egocentric updating. Finally, another disadvantage of real-environment paradigms lies in relatively difficult standardization and experimental manipulation—thus, it might be an option for future studies to take a hybrid approach by combining self-motion with the advantages of VR.

In conclusion, this study provides evidence of the precuneus being an important mediator for egocentric spatial updating in real-environment settings. The participants of the current study, while showing no deficits in navigational ability, performed significantly worse on our spatial updating paradigm after precuneus inhibition through cTBS compared to the sham condition, both in relation to translational (distance) and rotational (angle) errors. These findings are consistent with earlier studies involving patients with brain lesions or paradigms applying visual flow. Considering that our paradigm yielded high sensitivity and specificity values from a region of interest analysis with distance as parameter, it can be used as a sound base for the development of assessment tools for both healthy participants and various groups of patients.

## Figures and Tables

**Table 1:** Descriptive statistics and effect sizes for the main effects of stimulation (sham vs. cTBS) condition and movement (static vs. dynamic) condition (* *p <* 0.05, *** *p <* 0.001).

Parameter	Sham	cTBS	Diff. (CI)	Effect Size (d)
Distance	56.45 ± 17.85	65.12 ± 27.63	−8.67 (−15.40, −1.97)	−0.259 *
Angle	7.80 ± 3.34	8.44 ± 4.35	−0.65 (−1.94, 0.65)	−0.100
**Parameter**	**Static**	**Dynamic**	**Diff. (CI)**	**Effect size (d)**
Distance	49.26 ± 14.99	75.30 ± 21.54	−29.00 (−35.70, −22.30)	−0.856 ***
Angle	6.06 ± 2.54	10.18 ± 3.89	−4.12 (−5.42, −2.83)	−6.339 ***

**Table 2:** Descriptive statistics and effects sizes for the interaction effect between stimulation and movement condition on both distance and angular errors (** *p <* 0.01, *** *p <* 0.001).

Parameter	Stim. Condition	Static	Dynamic	Diff. (CI)	Effect Size (d)
Distance	**Sham**	48.83 ± 13.68	64.07 ± 18.57	−15.20 (−24.70, −5.75)	−0.322 **
	**cTBS**	43.7 ± 16.1	86.5 ± 18.5	−42.80 (−52.30, −33.35)	−0.904 ***
Angle	**Sham**	6.44 ± 2.24	9.15 ± 3.74	−2.71 (−4.54, −0.89)	−0.298 **
	**cTBS**	5.67 ± 2.82	11.21 ± 3.85	−5.54 (−7.36, −3.71)	−0.698 ***

## Data Availability

All data are stored at the FGW—Potsdam University and can only be obtained under special permission.
